# Risk of death by age and gender from CoVID-19 in Peru, March-May, 2020

**DOI:** 10.18632/aging.103687

**Published:** 2020-07-21

**Authors:** Cesar Munayco, Gerardo Chowell, Amna Tariq, Eduardo A. Undurraga, Kenji Mizumoto

**Affiliations:** 1 Centro Nacional de Epidemiología, Prevención y Control de Enfermedades, Peruvian Ministry of Health, Lima, Peru; 2 Department of Population Health Sciences, School of Public Health, Georgia State University, Atlanta, GA 30303, USA; 3 Escuela de Gobierno, Pontificia Universidad Católica de Chile, Santiago, Region Metropolitana, Chile; 4 Millennium Initiative for Collaborative Research in Bacterial Resistance, MICROB-R, Chile; 5 Graduate School of Advanced Integrated Studies in Human Survivability, Kyoto University Yoshida-Nakaadachi-cho, Sakyo-ku, Kyoto, Japan; 6 Hakubi Center for Advanced Research, Kyoto University, Yoshidahonmachi, Sakyo-ku, Kyoto, Japan

**Keywords:** COVID-19, Peru, risk of death, time-delay adjusted CFR, 2020

## Abstract

Peru implemented strict social distancing measures during the early phase of the epidemic and is now experiencing one of the largest CoVID-19 epidemics in Latin America. Estimates of disease severity are an essential indicator to inform policy decisions about the intensity and duration of interventions needed to mitigate the outbreak. Here we derive delay-adjusted case fatality risks (aCFR) of CoVID-19 in a middle-income country in South America.

We utilize government-reported time series of CoVID-19 cases and deaths in Peru stratified by age group and gender.

As of May 25, 2020, we estimate the aCFR for men and women at 10.8% (95%CrI: 10.5-11.1%) and 6.5% (95%CrI: 6.2-6.8%), respectively, whereas the overall aCFR was estimated at 9.1% (95%CrI: 8.9-9.3%). Our results show that senior individuals have been the most severely affected by CoVID-19, particularly men, with an aCFR of nearly 60% for those aged 80- years. We also found that men have a significantly higher cumulative morbidity ratio across most age groups (proportion test, p-value< 0.001), with the exception of those aged 0-9 years.

The ongoing CoVID-19 pandemic is generating a substantial mortality burden in Peru. Senior individuals, especially those older than 70 years, are being disproportionately affected by the CoVID-19 pandemic.

## INTRODUCTION

As of May 25, 2020, more than 5.5 million CoVID-19 cases and about340,000 deaths have been reported from almost every country and territory around the globe [1, 2]. The ongoing CoVID-19 pandemic has imposed a substantial burden on health systems, economies, and societies globally, and there are strong indicators pointing to a disproportionate impact on low- and middle-income countries [3–5]. Since its initial outbreak in China, the world has tracked the CoVID-19 pandemic proliferating across Europe and Asia, and later seeding hotspots in North America, the Middle East, and more recently in Latin America [6]. Brazil reported its first case on February 26, 2020 [7]. Neighboring countries started to report CoVID-19 cases in subsequent days; South America has registered more than 600,000 cases and 30,600 deaths as of May 24, 2020 [1]. Although many South American countries imposed strict control measures, including travel bans, school closures, and lockdowns early in the epidemic, the magnitude of their epidemics now rival those observed in European hotspots, with CoVID-19 cases and death counts increasing rapidly in the region [1, 5]. Other factors, including high poverty rates, informal economies, frail healthcare systems, insufficient medical supplies as well as inadequate water, sanitation, and hygiene infrastructure further exacerbate the health and socioeconomic impacts of the CoVID-19 pandemic [5, 8–10]. Governments in South America are now facing the social and economic consequences from SARS-COV-2 containment measures, while struggling to contain the rapidly expanding outbreaks of the deadly virus [9].

Peru, a country of about 30 million people, is experiencing one of the largest CoVID-19 epidemics in Latin America. With a rapidly rising case tally, Peru has reported almost 129,148 cases and 7660 deaths as of May 25, 2020 [11]. The majority (63%) of CoVID-19 cases have been confirmed in Lima, the capital of Peru [11]. The government of Peru initiated social distancing measures soon after the confirmation of the first imported case in Peru on March 6, 2020 [12]. The initial epidemic control measures included school closures on March 11, 2020 followed by the suspension of large gatherings and flights from Europe and Asia the next day. Subsequently the government declared a national emergency and closed its borders on March 16, 2020 [13]. Despite these forthcoming and swift control measures, untraced community transmission was reported by March 17, 2020, forcing the implementation of a night time curfew as of March 18, 2020 [13].

Estimates of the reproduction number from the early stage of the epidemic in Peru (March 2020) showed sustained transmission in Lima with a reproduction number R estimated at 2.3 (95% CI: 2.0, 2.5) [14]. Moreover, the 20-days ahead forecast for Lima suggested that the prompt social distancing measures had significantly slowed down the initial spread of the virus in the region [14]. Despite the implementation of non-pharmaceutical interventions in Peru, case and death counts have continued to rise rapidly. The crude case fatality risk (CFR), defined as the number of cumulative deaths and cases as of May 25, 2020, in Peru is estimated at 5.9%, which is in good agreement with the global crude CFR average of 6.3% [15]. Statistical analyses and mathematical models using data from Peru suggest that under current epidemic growth trends, the number of CoVID-19 infected individuals could surpass the country’s healthcare system capacity [16].

The clinical spectrum of CoVID-19 ranges from asymptomatic cases to clinical conditions characterized by respiratory failure, to multiorgan and systemic manifestations which can cause death [17–19]. The SARS-CoV-2 virus is more likely to generate severe disease among individuals ≥60 years of age, especially those with preexisting medical conditions that include heart disease, lung disease, diabetes or cancer [20]. Further, CoVID-19 associated deaths occur more frequently (about 80% of total deaths) in persons aged ≥65 years based on data from the USA, and consistent with data from China indicating that >80% CoVID-19 deaths occur among persons aged ≥60 years [21]. Moreover, a higher crude fatality risk has been reported among men (2.8% for men versus 1.7% for women) in China [22]. Age adjusted CFR estimates from Peru can be useful to gauge the mortality impact of the pandemic and assess whether the severity patterns are consistent in the South America, a region with fragmented health systems, vast inequality, and high poverty rates.

CFR is a key epidemiological metric that quantifies the severity of an epidemic [23], aiding public health officials assess the type and intensity of interventions that need to be implemented to mitigate its impact [24]. However, it becomes challenging to estimate CFR during an epidemic as CFR estimates are sensitive to right censoring of the data that occurs because of the time lag between the symptoms onset and death [25–27]. Moreover, under-reporting of cases because mild or asymptomatic cases can go undetected by disease surveillance systems also overestimates CFR [25, 28], while CFR estimates by subgroup are less prone to sampling bias and help identify the most vulnerable subpopulations. For comparison, the infection fatality risk (IFR) is calculated by the ratio of cumulative deaths over the cumulative number of infected individuals.

Given the importance of timely CFR estimates for public health decision making, we provide real-time estimates of adjusted age-specific CFR during the CoVID-19 epidemic in Peru, through May 25, 2020 to assess the pandemic’s severity variation in this southern hemisphere setting, which helps pinpoint the most vulnerable segments of the population and tailor public health interventions.

## RESULTS

**Figure 1:**
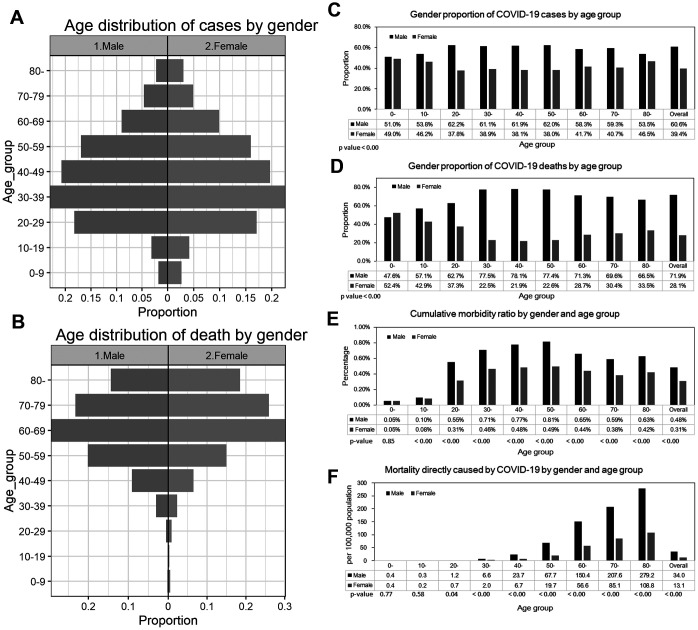
**Epidemiological characterization of CoVID-19 in Peru, as of May 25, 2020.** (**A**) Age distribution of reported cases by gender, (**B**) Age distribution of reported deaths by gender. (**C**) Gender proportion of CoVID-19 cases by age group, (**D**) Gender proportion of CoVID-19 deaths by age group, (**E**) Cumulative morbidity risk by gender and age group, (**F**) Mortality directly caused by CoVID-19 by gender and age group.

As of May 25, a total of 129,148 cases and 7,660 deaths due to CoVID-19 have been reported by the Ministry of Health, Peru. Among men, reported cases were mostly observed among individuals aged 30-39 years (23.1%), followed by those aged 40-49 years (20.8%), and those aged 20-29 years (18.3%). In contrast, most deaths were reported among those aged 50 years and above, especially among men aged 60-69 (29.3%) followed by those aged 70-79 (23.2%), aged 50-59 years (20.1%), and aged 80 years and above (14.3%). (Table 1, Figure 1A, 1B). Data show a similar pattern for women. The majority of reported cases occur in females aged 20-69 years, and the majority of reported deaths occur among women aged 50 years or more. More specifically, most reported cases occur among women aged 30-39 (22.6%), followed by women aged 40-49 (19.7%), and 50-59 year olds (16.0%). In contrast, most deaths are reported among those aged 60-69 (30.2%), followed by women aged 70-79 (25.9%), and lastly, women aged 80 years and above (18.4%). Regarding CoVID-19 mortality per 100,000 population, seniors (individuals >70 years of age) were the most affected age group; mortality burden per 100,000 is 279.2 among men aged 80 years and above, and 207.6 among men aged 70-79 years. For women of 80 years of age or more mortality is 108.8 and 85.1 for women aged 70-79 years (Table 1, Figure 1F).

**Table 1:** Distribution of the cases by sex and age groups, as of May 25, 2020.

**Age group**	**Men**					**Women**			
	**Cases (%)**	**Deaths (%)**	**cCFR (%)**	**Mortality per 100,000 population**		**Cases (%)**	**Deaths (%)**	**cCFR(%)**	**Mortality per 100,000 population**
All	78264	5508	7.0%	34.0		50884	2152	4.2	13.1
	(100)	(100)				(100)	(100)		
0-9	1416	10	0.7	0.4		1362	11	0.8	0.4
	(1.8)	(0.2)				(2.7)	(0.5)		
10-19	2475	8	0.3	0.3		2128	6	0.3	0.2
	(3.2)	(0.1)				(4.2)	(0.3)		
20-29	14306	32	0.2	1.2		8707	19	0.2	0.7
	(18.3)	(0.6)				(17.1)	(0.9)		
30-39	18052	169	0.9	6.6		11487	49	0.4	2.0
	(23.1)	(3.1)				(22.6)	(2.3)		
40-49	16258	499	3.1	23.7		10005	140	1.4	6.7
	(20.8)	(9.1)				(19.7)	(6.5)		
50-59	13274	1107	8.3	67.7		8124	323	4.0	19.7
	(17.0)	(20.1)				(16.0)	(15.0)		
60-69	7034	1615	23.0	150.4		5023	649	12.9	56.6
	(9.0)	(29.3)				(9.9)	(30.2)		
70-79	3620	1279	35.3	207.6		2488	558	22.4	85.1
	(4.6)	(23.2)				(4.9)	(25.9)		
80 -	1769	789	44.6	279.2		1536	397	25.8	108.8
	(2.3)	(14.3)				(3.0)	(18.4)		

The gender proportions of reported cases by age groups are presented in Figure 1C and Figure 1D. The proportion of cases among men is higher than 50% across all age groups (χ^2^ test, p-value<0.001). Similarly, the proportion of male deaths is also higher than 50% except for those aged 10-19 years (χ^2^ test, p-value<0.001). Cumulative morbidity ratio by gender and age group is presented in Figure 1E, indicating that cumulative morbidity ratio among men is higher than women across all age groups (proportion test, p-value < 0.001) except for individuals aged 0-9 years (proportion test, p-value =0.85). Figure 1F illustrates the mortality per 100,000 population directly caused by CoVID-19 by gender and age group. Mortality is higher than among females aged 20 years and above (proportion test, p-value <0.05), and it is not significantly different among those aged 0-19 years.

**Figure 2:**
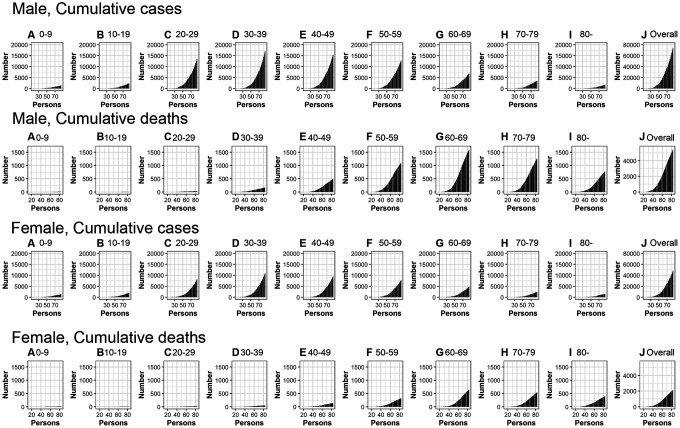
**Temporal distribution of cases and deaths by age group due to CoVID-19, March-May 2020, Peru.** Top: Male, cumulative cases, Second top: Male, cumulative cases, Second bottom: Female, cumulative cases, Bottom: Female cumulative deaths (**A**) aged 0-9, (**B**) aged 10-19, (**C**) aged 20-29, (**D**) aged 30-39, (**E**) aged 40-49, (**F**) aged 50-59, (**G**) aged 60-69, (**H**) aged 70-79, (**I**) aged 80- and (**J**) Overall (all age groups). Day 1 corresponds to March 1^st^ in 2020.

Figure 2 shows the cumulative cases and deaths of CoVID-19 by age group for males and females (A through J) over time. The figure suggests cumulative deaths increases after an increase in cumulative cases. The growth curve for overall cumulative cases (all age groups) for men and women appears to increase exponentially until around day 60 (April 29^th^, 2020), while exponential growth in cumulative deaths overall (all age groups) for men and women appears to occur until around day 70 (May 9^th^, 2020).

**Figure 3:**
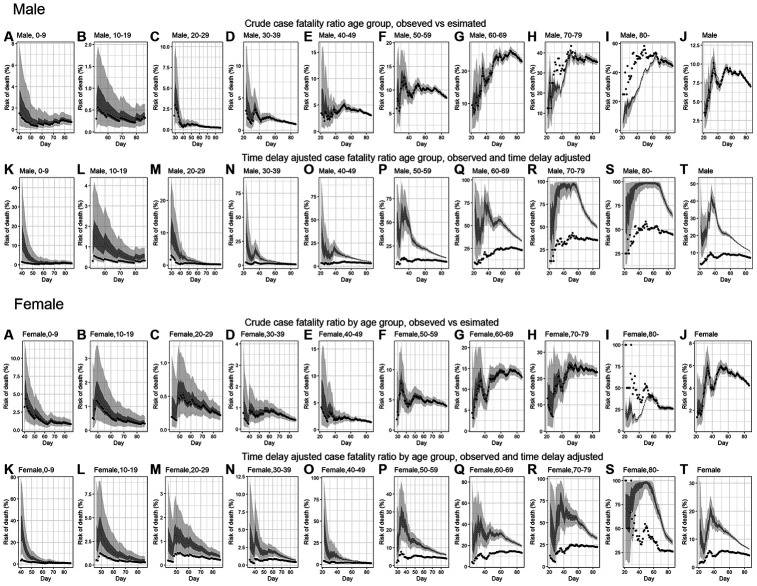
**Temporal variation of male and female risk of death by age group caused by CoVID-19, March-May 2020, Peru.** Upper two rows; Male risk of deaths, Lower two rows; Female risk of deaths. Observed and posterior estimated of crude case fatality risk of (**A**) aged 0-9, (**B**) aged 10-19, (**C**) aged 20-29, (**D**) aged 30-39, (**E**) aged 40-49, (**F**) aged 50-59, (**G**) aged 60-69, (**H**) aged 70-79, (**I**) aged 80-, (**J**) all age groups and time-delay adjusted case fatality risk of (**K**) aged 0-9, (**L**) aged 10-19, (**M**) aged 20-29, (**N**) aged 30-39, (**O**) aged 40-49, (**P**) aged 50-59, (**Q**) aged 60-69, (**R**) aged 70-79, (**S**) aged 80-, (**T**) all age groups. Day 1 corresponds to March 1^st^ in 2020. Black dots show crude case fatality risk, and light and dark indicates 95% and 50% credible intervals for posterior estimates, respectively.

Figure 3 illustrates observed and model based posterior estimates of the crude CFR by age group (A-J) and time-delay adjusted CFR by age group (K-T) for men and women. Black dots show crude case fatality risks, and light and dark indicate 95% and 50% credible intervals (CrI) for posterior estimates, respectively.

Overall, our model based crude CFR fitted the observed data well, except for individuals aged 80 years and above, probably influenced by low reporting rate/ascertainment bias of cases at an early stage. Crude CFR for most of age groups increased at the early stage of the epidemic, peaked amidst the outbreak day 34 (April 3^rd^, 2020) and followed a decreasing trend turning into an almost flat curve.

Overall, our model-based posterior estimates for the time-delay adjusted CFR are substantially higher than the crude observed CFR. These estimates fluctuated at the early stage of the epidemic and then followed a decreasing trend.

**Figure 4:**
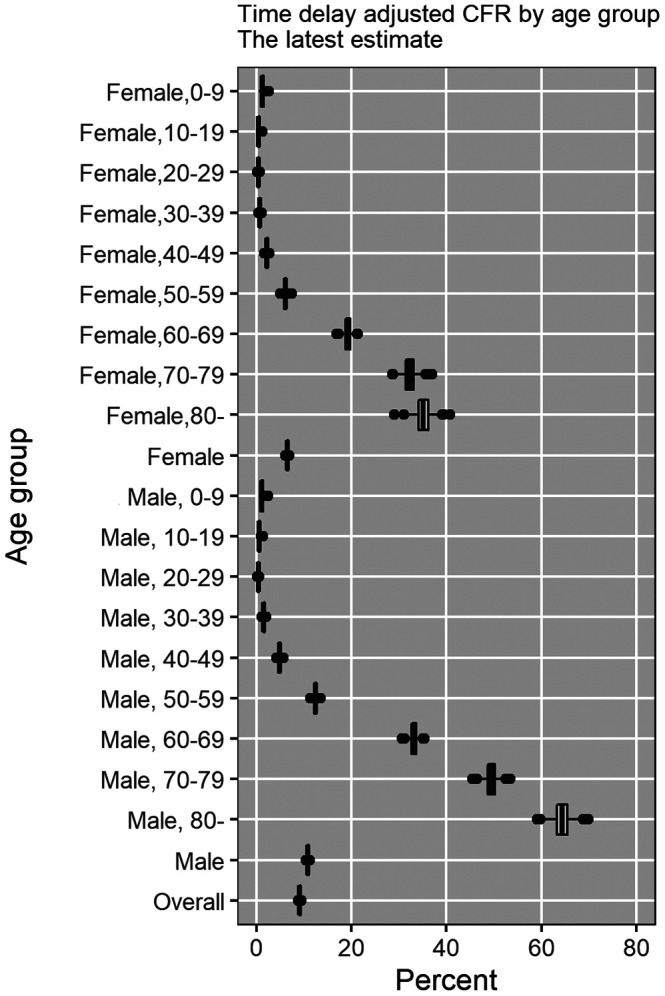
**Most recent estimates of time-delay adjusted risk of death caused by CoVID-19 by age group and gender, March-May 2020, Peru.** Distribution of time-delay adjusted risk of death from the latest estimates (May 25, 2020) is presented. Top to bottom: female aged 0-9, female aged 10-19, female aged 20-29, female aged 30-39, female aged 40-49, female aged 50-59, female aged 60-69, female aged 70-79, female aged 80 and over, female overall.

The most recent estimates, as of May 25, 2020, of the time-delay adjusted CFR for men and women are 10.8% (95%CrI: 10.5-11.1%) and 6.5% (95%CrI: 6.2-6.8%), respectively, while overall national estimate is 9.1% (95%CrI: 8.9-9.3%) (Figure 4 and Table 2). Among men, senior citizens appear to be severely affected; the adjusted CFR is 33.1% (95%CrI: 31.7-34.6%) for men aged 60-69 years, 49.4% (95%CrI: 47.3-51.6%) for those aged 70-79 years, and 64.3% (95%CrI: 60.9-67.8%) for those 80 years old and above. We observe a similar pattern for women. The adjusted CFR is 19.2% (95%CrI: 17.9-20.6%) for women aged 60-69 years, 32.2% (95%CrI: 29.9-34.7%) for those aged 70-79 years, and 35.1% (95%CrI: 32.1-38.1%) for women aged 80 years old or more.

**Table 2:** Summary results of time-delay adjusted case fatality risk of CoVID-19 in each age group in Peru, 2020 as of May 25, 2020.

**Age group**	**Gender**	**Latest estimate**	**Range of median estimates**	**Crude case fatality rate**
Overall		9.1% (95%CrI^a^: 8.9-9.3%)	9.1-32.0%	5.9% (95%CI^b^: 5.8-6.1%)
				7660/129148 ^c^
Male		10.8% (95%CrI: 10.5-11.1%)	10.8-42.3%	7.0% (95%CI: 6.9-7.2%)
				5508/78264
Female		6.5% (95%CrI: 6.2-6.8%)	6.4-20.0%	4.2% (95%CI: 4.1-4.4%)
				2152/50884
0-9	Male	1.1% (95%CrI: 0.5-1.8%)	1.0-13.3%	0.7% (95%CI:0.3-1.3%)
				10/1416
	Female	1.2% (95%CrI: 0.7-2.0%)	1.2-31.4%	0.8% (95%CI: 0.4-1.4%)
				11/1362
10-19	Male	0.5% (95%CrI: 0.3-1.0%)	0.4-1.6%	0.3% (95%CI: 0.1-0.6%)
				8/2475
	Female	0.5% (95%CrI: 0.2-0.9%)	0.4-3.8%	0.3% (95%CI: 0.1-0.6%)
				6/2128
20-29	Male	0.4% (95%CrI: 0.2-0.5%)	0.4-10.0%	0.2% (95%CI: 0.2-0.3%)
				32/14306
	Female	0.4% (95%CrI: 0.2-0.6%)	0.4-1.3%	0.2% (95%CI: 0.1-0.3%)
				19/8707
30-39	Male	1.5% (95%CrI: 1.3-1.7%)	1.5-32.3%	0.9% (95%CI: 0.8-1.1%)
				169/18052
	Female	0.7% (95%CrI: 0.5-0.9%)	0.9-4.4%	0.4% (95%CI: 0.3-0.6%)
				49/11487
40-49	Male	4.8% (95%CrI: 4.4-5.2%)	4.8-34.7%	3.1% (95%CI: 2.8-3.3%)
				499/16258
	Female	2.2% (95%CrI: 1.8-2.5%)	2.2-44.6%	1.4% (95%CI: 1.2-1.6%)
				140/10005
50-59	Male	12.4% (95%CrI: 11.7-13.1%)	12.4-60.0%	8.3% (95%CI: 7.9-8.8%)
				1107/13274
	Female	6.0% (95%CrI: 5.4-6.7%)	7.5-27.7%	4.0% (95%CI: 3.6-4.4%)
				323/8124
60-69	Male	33.1% (95%CrI: 31.7-34.6%)	33.1-77.8%	23.0% (95%CI: 22.0-24.0%)
				1615/7034
	Female	19.2% (95%CrI: 17.9-20.6%)	19.2-40.9%	12.9% (95%CI: 12.0-13.9%)
				649/5023
70-79	Male	49.4% (95%CrI: 47.3-51.6%)	48.7-97.8%	35.3% (95%CI: 33.8-36.9%)
				1279/3620
	Female	32.2% (95%CrI: 29.9-34.7%)	24.1-74.9%	22.4% (95%CI: 20.8-24.1%)
				558/2488
80-	Male	64.3% (95%CrI: 60.9-67.8%)	64.3-98.9%	44.6% (95%CI: 42.3-47.0%)
				789/1769
	Female	35.1% (95%CrI: 32.1-38.1%)	35.1-98.3%	25.8% (95%CI: 23.7-28.1%)
				397/1536

^a^ 95%CrI: 95% credibility intervals (CrI), ^b^ 95%CI: 95% confidence interval, ^c^ Cumulative cases over cumulative deaths

## DISCUSSION

This study estimates the time-delay adjusted CFR by age group for the ongoing CoVID-19 epidemic in Peru. The crude CFR varies across countries due to differences in testing and timing of tests [29]. The results from our analysis show that the CoVID-19 epidemic in Peru disproportionately impacts senior individuals, especially those who are 70 years of age or older, consistent with CFR estimates obtained from recent studies conducted in China [30, 31], Chile [32], and Italy [33, 34]. This pattern suggests that an aging population could aggravate the fatality impact of CoVID-19, influenza and respiratory syncytial virus [32], as was probably an important factor for its high impact in Italy [33, 34]. While the population in Lain America, including Peru, is aging at a rapid rate, still a relatively small percentage of the population in the region are older than 65 years of age [35]. Hence, the age structure in the region could favor a lower overall CFR than would be expected otherwise with a relatively older population, as in other regions.

Our estimate of adjusted CFR among men (10.8% (95%CrI: 10.5-11.1%)) is 1.7-fold higher than the estimated adjusted CFR for women (6.5% (95%CrI: 6.2-6.8%)), consistent with the estimates given in ref [37]. Men aged 80 years or older have an estimated adjusted CFR as high as 64.3% (95%CrI: 60.9-67.8%), 58-fold higher than our estimates for men aged 0-9, and 1.3-fold higher than our estimates for men aged 70-79. Similarly, the adjusted CFR estimates for women of aged 80 years or older are as high as 35.1% (95%CrI: 32.1-38.1%), 29-fold higher than the estimates obtained for female aged 0-9 and 1.1-fold higher than the estimates obtained for female aged 70-79, consistent with recent findings in Chile [32]. In comparison, a study conducted in China, reported much lower estimates of CFR for individuals >80 years of age (13.4%) [31].

An upward trend in the crude CFR for overall population suggests the disease transmission may be spreading to more vulnerable populations. The majority of social distancing measures in Peru were implemented between March 11-March 18, 2020. However, since 72.4% of the economically active population works in informal jobs, which are concentrated in the poorest areas of the country, compliance with government mitigation strategies can be challenging despite the government’s efforts to support the population [37]. Another factor possibly contributing to the upward trend in crude CFR may be an increase in unreported cases due to saturated testing capacity [29]. However, since Peru’s testing capacity has substantially increased since the beginning of the outbreak, going from >0.01 test per 1000 population to 0.09 per 1000 in May 22 [15], and the positivity rate estimated at 8.6% for March, 2020, this seems an unlikely cause. In Peru, about 85% of ICU beds with ventilators are currently occupied by patients [37], therefore our present estimates are not affected by excess deaths due to health care demand exceeding health care capacity. However, as the epidemic continues to expand, healthcare capacity may be reached in the short term [37]. Furthermore, the results show an increasing trend in crude CFR around day 45 (May 14^th^, 2020), probably reflecting the exponential increase of cumulative cases around day 40 (May 9^th^, 2020).

The downward trend in the adjusted CFR at the early stage may indicate the existence of a reporting delay and the shift of the outbreak to a less vulnerable segment of the population. In particular, the observed differences in estimates between the crude CFR and adjusted CFR can be attributed to the time-delay that is assumed fixed during the course of the epidemic.

The relatively small proportion of males (53.5%) among CoVID-19 cases in the individuals aged 80 years and above can be attributed to the relatively small male population size for that age group; with men comprising only 1.7% of the population >80 years of age in Peru, consistent with estimates for Chile [32]. As higher mortality among male has been reported in China and the U.S. [38], additional data on deaths stratified by gender provides the opportunity to examine the CFR by gender and age.

Several studies documenting the IFR of CoVID-19 have been reported based on an observational study [39], modeling studies [31, 40] and serological studies [41, 42]. While IFR estimates may be more realistic indicators compared to estimates derived from observed cases alone [43, 44], the external validity of these serological studies, e.g., whether the results can be applied to the generalized population in the region where they are performed, needs to be closely examined, as pointed out elsewhere [40, 45, 46]. In particular, to derive IFR estimates, prevalence, the cumulative number of infected people, is estimated based on the result of serological studies. Then, the cumulative number of deaths in the region is divided by the estimated cumulative number of infected individuals.

Indeed, serological studies based on blood donors and outpatients/hospitalized patients will easily lead to overestimation and underestimation, respectively, because the number of infected individuals is expected to be lower among the blood donors and higher among the outpatients/hospitalized patients. In contrast, the death risk derived from the CFR is less affected by the sampling bias and a convenient indicator to identify the vulnerable subpopulations, especially focusing on a single country with relatively uniform testing capacity across the population.

Our study has at least two limitations. First, our estimates are probably overestimated, due to the effect of under reporting rates and ascertainment rates, as has been underscored in other studies [25, 27, 47]. But a recently enhanced testing capacity in Peru is expected to mitigate these effects, and an ongoing mass serological study will provide data to generate more accurate estimates of the death risk. Second, adjusted CFR, especially among seniors, has displayed fluctuations, highlighting the importance of focusing on sub-group analyses. Additional information such as line lists that include related risks including information on underlying diseases may help to identify subgroups with elevated risks.

## CONCLUSIONS

The CoVID-19 pandemic is imposing a large death toll in Peru. Senior individuals, especially those who are older than 70 years of age, are being disproportionately affected by the CoVID-19 pandemic, particularly elderly men. CFR was as high as 64.3% (95%CrI: 60.9-67.8%) for men aged 80 older, 58-fold higher than our estimates for men aged 0-9. The overall adjusted CFR in Peru is estimated to be higher than in other countries, which is worrying, particularly because healthcare demand has not yet exceeded capacity, but probably will do in the coming weeks. The relatively younger age structure in Latin America may help ameliorate the overall CFR than would otherwise be expected with an older age structure in the population.

## MATERIALS AND METHODS

### Data

We obtained daily cumulative numbers of reported laboratory confirmed CoVID-19 cases and deaths stratified by age group and gender through May 25, 2020. Different age groups had different starting times, which correspond to the day when death was reported. Confirmed CoVID-19 cases were retrieved from three surveillance systems: a) national surveillance system (confirmed and suspected cases based on a case definition), b) Netlab system (molecular test) and c) SICOVID system (rapid serological test). CoVID-19 deaths were obtained from two surveillance systems: a) national surveillance system (confirmed and suspected deaths based on a case definition) and b) Vital statistics system (National System of mortality -SINADEF- which is an online system that keeps track of death certificates) [48]. A suspected case presents with acute respiratory infection and with two or more of the following symptoms (cough, sore throat, respiratory distress, nasal congestion or fever), close contact with a CoVID-19 case within 14 days of symptoms onset, or people who live or traveled to cities with community transmission of SARS-CoV-2 within 14 days of symptoms onset. On the other hand, the definition of confirmed cases is a suspected case with a positive lab test. [49].

Population size by age, group, and gender in 2020 were retrieved from the Ministry of Health in Peru [50]**.**

### Statistical analysis

The crude CFR is defined as the number of cumulative deaths over the number of cumulative cases. For the estimation of CFR in real time, we employed the delay from hospitalization to death, h_*s*_, which is assumed to be given by h_*s*_ = H(s) – H(s-1) for s>0 where H(s) is a cumulative density function of the delay from hospitalization to death and follows a gamma distribution with mean 10.1 days and SD 5.4 days, as given in ref, Mizumoto and Chowell [24]. Let π_*a,ti*_ be the time-delay adjusted case fatality risk on reported day t_*i*_ in area *a*, the likelihood function of the estimate $\pi_{a,t_{i}}$ is given by equation:

$$\begin{array}{l} L(\pi_{a,t_{i}};c_{a,t},D_{a,t})\\ =\underset{t_{i}}{\prod }\left(\begin{array}{c} \overset{t_{i}}{\underset{t=1}{\sum }}c_{a,t}\\ D_{a,t_{i}} \end{array}\right){\left(\pi_{a,t_{i}}\frac{\sum_{t=2}^{t_{i}}\sum_{s=1}^{t-1}c_{a,t-s}h_{s}}{\sum_{t=1}^{t_{i}}c_{a,t}}\right)}^{D_{a,t_{i}}}\\ {\left(1-\pi_{a,t_{i}}\frac{\sum_{t=2}^{t_{i}}\sum_{s=1}^{t-1}c_{a,t-s}h_{s}}{\sum_{t=1}^{t_{i}}c_{a,t}}\right)}^{\sum_{t=1}^{ti}c_{a,t}-D_{a,t_{i}}} \end{array}$$

where c_*a,t*_ represents the number of new cases with reported day t in area *a*, and $D_{a,t_{i}}$ is the cumulative number of deaths until reported day t_*i*_ in area *a* [51, 52]. Among the cumulative cases with reported day *t* in area *a*, $D_{a,t_{i}}$ have died and the remainder have survived the infection. The contribution of those who have died with biased death risk is shown in the middle parenthetical term and the contribution of survivors is presented in the right parenthetical term. We assume that $D_{a,t_{i}}$ is the result of the binomial sampling process with probability $\pi_{a,t_{i}}$.

We used a Monte Carlo Markov Chain (MCMC) method in a Bayesian framework to estimate model parameters. We evaluated the convergence of MCMC chains using the potential scale reduction statistic [53, 54]. Estimates and 95% credibility intervals for these estimates are based on the posterior probability distribution of each parameter and samples drawn from the posterior distributions. All statistical analyses were conducted in R version 3.6.1 (R Foundation for Statistical Computing, Vienna, Austria) using the ‘rstan’ package.
